# Identification of Genetic Interaction with Risk Factors Using a Time-To-Event Model

**DOI:** 10.3390/ijerph14101228

**Published:** 2017-10-15

**Authors:** Mariza de Andrade, Sebastian M. Armasu, Bryan M. McCauley, Tanya M. Petterson, John A. Heit

**Affiliations:** 1 Division of Biomedical Statistics and Informatics, Department of Health Sciences Research, Mayo Clinic, Rochester, MN 55905, USA; armasu.sebastian@mayo.edu (S.M.A.); mccauley.bryan@mayo.edu (B.M.M.); petterst@mayo.edu (T.M.P.); 2 Division of Epidemiology, Department of Health Sciences Research; Mayo Clinic, Rochester, MN 55905, USA; Heit.John@mayo.edu; 3 Department of Cardiovascular Diseases, Mayo Clinic, Rochester, MN 55905, USA

**Keywords:** genome-wide association study, genetic variation, pregnancy complications, risk factors, venous thromboembolism

## Abstract

Background: Certain diseases can occur with and without a trigger. We use Venous Thromboembolism (VTE) as our example to identify genetic interaction with pregnancy in women with VTE during pre- or postpartum. Pregnancy is one of the major risk factors for VTE as it accounts for 10% of maternal deaths. Methods: We performed a whole genome association analysis using the Cox Proportional Hazard (CoxPH) model adjusted for covariates to identify genetic variants associated with the time-to-event of VTE related to pre- or postpartum during the childbearing age of 18–45 years using a case-only design in a cohort of women with VTE. Women with a VTE event after 45 years of age were censored and contributed only follow-up time. Results: We identified two intragenic single nucleotide polymorphisms (SNPs) at genome-wide significance in the *PURB* gene located on chromosome 7, and two additional intragenic SNPs, one in the *LINGO2* gene on chromosome 9 and one in *RDXP2* on chromosome X. Conclusions: We showed that the time-to-event model is a useful approach for identifying potential hazard-modification of the genetic variants when the event of interest (VTE) occurs due to a risk factor (pre- or post-partum).

## 1. Introduction

Venous thromboembolism (VTE), consisting of deep vein thrombosis (DVT) and its complication pulmonary embolism (PE), is a common and potentially fatal disease, with an incidence of about 200 per 100,000 women-years among pregnant or postpartum women, and accounting for approximately 10% of all maternal deaths [1,2,3,4,5]. Family and twin studies suggest that VTE is highly heritable (*h*^2^ = 0.62) and likely results from multi-genic action as well as environmental exposures [6,7,8]. Approximately 80% of VTE events during pregnancy are DVT and 20% are PE [9]. It is well-known that hyper-coagulation, vascular damage, and venous stasis all occur in pregnancy, resulting in a relative risk of 4.3 with a 95% confidence interval (CI) from 3.5 to 5.2 for VTE in pregnant or postpartum women compared with non-pregnant women [5,10]. Known risk factors for pregnancy-related VTE are age greater than 35 years, obesity (body mass index > 30 kg/m^2^), multi-parity, and personal or family history of VTE or thrombophilia [11,12,13]. Since one-quarter of PE patients present as sudden death [7], the incidence of VTE must be reduced in order to improve survival. However, VTE incidence remained relatively unchanged—at about 1 per 1000 person-years—between 1981 and 2010 [10,14,15]. We suspect that the failure to reduce VTE incidence during pregnancy and postpartum is largely due to our limited understanding of which individuals should be targeted for prophylaxis. Despite multiple studies of the clinical and genetic risk factors for VTE [16,17], the same genetic variants have been identified (Factor V Leiden, Prothrombin G20210A, and ABO non-O) along with, depending on the sample size, a handful of new single nucleotide polymorphisms (SNPs). Furthermore, no VTE study has explored the contribution of genetic variants to the hazard of an incident, pregnancy-related VTE at a genome-wide level. Novel and replicated genetic VTE risk factors identified by genome-wide association studies (GWAS) together account for only a small percentage of VTE heritability (estimated *h*^2^ ~ 0.5–0.6 using VTE families) [6]. In our candidate gene case-control study, we showed that the joint attributable risk for VTE of three main genetics risk factors (Factor V Leiden mutation, Prothrombin G20210A, and ABO blood type non-O) was 0.40 [18]. Thus, there is a high likelihood that the missing link can be explained by the combination between genetic predisposition and environmental risk factors. While several exposures (e.g., surgery, trauma/fracture, and pregnancy/puerperium) are strongly associated with VTE, these exposures have poor predictive value for the individual [19]. In this paper, we present results for a genome-wide single SNP association with the risk of incident, pregnancy-related VTE in 634 VTE female cases, using the Cox Proportional Hazard Model (CoxPH). In GWAS of VTE risk, CoxPH is not as commonly used as the classical logistic regression model that does not take into account the time-to-event.

## 2. Materials and Methods

### 2.1. Study Design, Population, and Genotyping

We report a genome-wide investigation of genetic susceptibility variants for the risk of incident, pregnancy-related VTE in females of non-Hispanic European ancestry that were referred to the Thrombophilia Center at Mayo Clinic. These women are from the Mayo Clinic VTE Study [18,20]. The present study uses only VTE female cases. Only 634 females with VTE, pregnancy information available for the age range between 18 and 45 years, with genome-wide genotype data and 1000G imputed data using Impute2 software (University of Oxford, Oxford, UK ), were analyzed [21,22]. One female was excluded due to an invalid clot date. From the remaining 633 females, 277 had their VTE event during the childbearing age of 18 to 45 years. The minimum age for enrollment in the study was 18 years old. All subjects gave their informed consent for inclusion before they participated in the study. The study was conducted in accordance with the Declaration of Helsinki, and the protocol “Genome Wide Association of Venous Thromboembolism” was approved by the Mayo Clinic Institutional Board Review ID 08-002741 on 24 October 2008, and it is revised and approved yearly. The participants’ information, such as age, date of VTE, sex, body mass index (BMI), incidence of stroke, incidence of myocardial infarction (MI), and genotype, are available in the database of Genotypes and Phenotypes (dbGaP) under the accession number: phs000289.v2.p1, and the 1000G imputed data are available as part of the electronic Medical Records and Genomics (eMERGE) network under the accession number phs00360.v3.p1 [23]. In this analysis, we included only imputed SNPs with an imputation quality greater than 0.8 and a minor allele frequency (MAF) higher than 0.005 in the association analysis.

### 2.2. Statistical Methods

Our objective is to identify associations between genetic variants and risk of pregnancy-related VTE. A window of nine months prior to delivery and three months after delivery was used to define the pregnancy and postpartum period. In our data, all of the 277 VTE events are the first VTE (incident VTE) during the age range of 18–45 years. We censored the cases after 45 years old, since they were no longer able to be pregnant and no longer could have a pregnancy-related VTE. Thus, in our statistical model, pregnancy (9 months) or pregnancy/post-partum (12 months) is a time-dependent risk factor. Then, pregnancy or pregnancy/postpartum is a start-stop time variable up to the age of 45 years. Thus, we are interested in answering the following questions: Does a previous pregnancy contribute to the hazard of an incident pregnancy-related VTE? Are the genetic variants associated with incident pregnancy-related VTE the same genetic variants associated with incident VTE?

We used the Anderson–Gill (counting process) formulation of the CoxPH model with time on the age scale. The period of risk for an event, described as a VTE during pregnancy or postpartum, was from age 18 to 45. No one with a VTE before age 18 was included in this study; those women with a VTE after the age of 45 were censored at age 45. Any women with a VTE between 18 and 45 years of age, unrelated to pregnancy/postpartum, were censored at the time of the unrelated VTE. Subjects were also censored if death occurred between the ages of 18 and 45. Since pregnancy is a well-known risk factor for VTE [24,25], we wanted to examine the relationship of pregnancy accounting for each episode, and treated each episode as a time-dependent covariate. We applied the Schoenfeld residuals test to check the proportional hazard assumption for pregnancy, and used the start-stop counting process style of input within the Cox regression framework in the R library ‘survival’ and the ‘coxph’ function to identify genetic variants associated with time to VTE event due to pregnancy, adjusting for pregnancy, stroke/myocardial infarction, and state of residence [26].

### 2.3. Complementary Approaches to Validate the Results

Since we do not have a replication set with a similar design to ours, we employed two validation strategies for the genome-wide significant SNPs. The first strategy was an internal cross-validation strategy [27]. For the CoxPH model, the replication sets were created by sorting the CoxPH residuals from the model with no covariates and counting off groups of 10, i.e., the first residual goes in replication set 1, the 11th residual goes in replication set 1, the 2nd residual goes in replication set 2, and so on. This created N sets balanced in terms of the time-to-event variables. Then, there are K ways of choosing 2 sets of the 10 for the 20% test set, resulting in the 80% and 20% data sets having K combinations each. The second strategy was a fixed-effect meta-analysis approach, where we divided the samples into two datasets using the same strategy of sorting the residuals from the model with no covariates, as described above: the first set as the discovery set and consisting of 70% of the samples, and the second set as the replication set and consisting of 30% of the samples. A Woolf’s test of homogeneity of hazard ratios (HRs) between the discovery and validation sets was performed to assess whether the distribution of HRs between the two sets is compatible with a common HR [28]. For each set, we use the CoxPH model described in Statistical Methods. All of the statistical and validation analyses were performed using R version 2.15 [29].

### 2.4. Whole Blood Gene Expression

Whole blood expressions were available for adult Mayo Clinic patients of non-Hispanic European ancestry with objectively confirmed VTE (*n* = 58) and controls (*n* = 25), as part of a gene expression study previously reported and described [30,31], and they do not overlap with our present study. From those, 5 VTE cases and 12 controls were removed due poor quality control, resulting in 53 cases and 13 controls ready for analysis. From those, 30 were females, 22 with VTE and 8 controls.

## 3. Results

### 3.1. Genome-Wide Association Using CoxPH Model and 1000G Imputed Data

**Figure 1:**
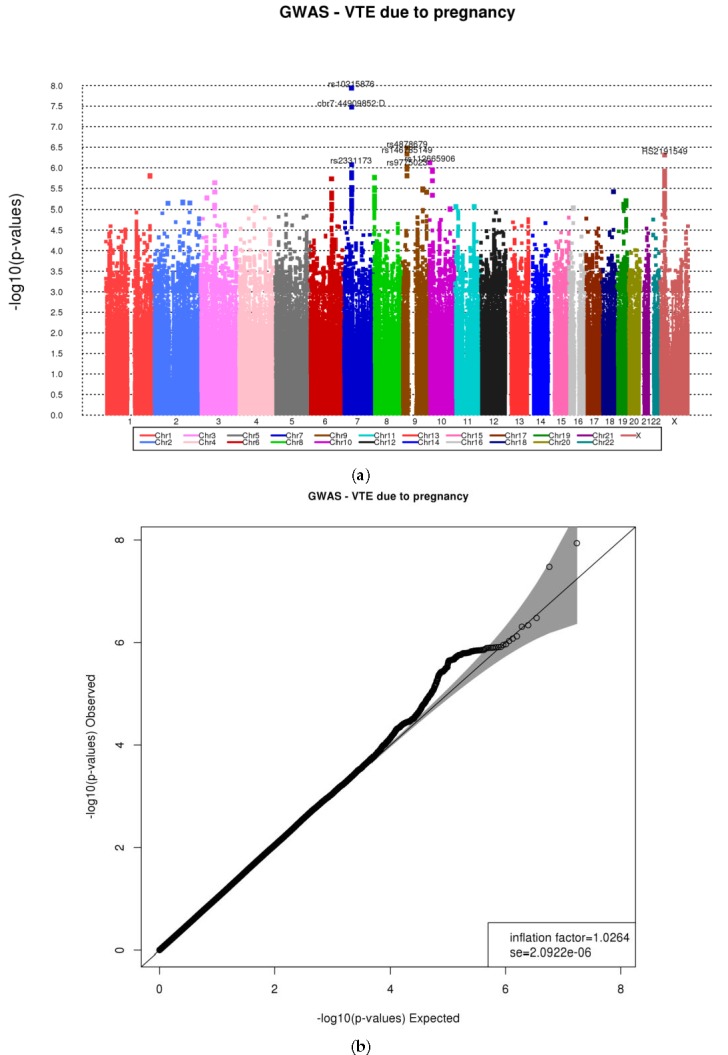
(**a**) Manhattan plot for the genome-wide association (GWA) for pregnancy-related venous thromboembolism (VTE) with the two significant single nucleotide polymorphisms (SNPs) within the expected level of significance; (**b**) QQ plot for the genome-wide association (GWA) for pregnancy-related VTE with the two significant SNPs within the expected level of significance, the gray area indicating that they are not spurious results. GWAS: genome-wide association study.

From the 634 VTE female cases, 277 had their VTE event between 18 and 45 years of age; the remaining 357 had their VTE event after age 45. The distribution of VTE females by pregnancy, oral contraceptive (OC), hormone replacement therapy (HRT), and family history of VTE is shown in Table 1a. The HR for pregnancy, OC, and HRT are highly significant with the outcome, with the exception of family history. A detailed description of the 277 with VTE between age 18 and 45 is depicted in Table 1b: 172 were never pregnant, 75 had a pregnancy between the ages of 18 and 45 but the VTE event did not occur at the time of pregnancy, and 30 had a VTE at the time of pregnancy (event). Additional information on the timing of the pregnancy and VTE can be found in Table 2. The proportional hazards assumption for pregnancy was assessed using the Schoenfeld residuals test and it held in the data (*p* = 0.73). The risk factors pregnancy (HR = 3.19, 95% CI (2.16–4.71), *p* = 3.5 × 10^−7^) and Stroke/MI (HR = 0.49, 95% CI (0.33–0.74), *p* = 1.65 × 10^−4^) were found to have a significant association with the pregnancy-related VTE event (Table 3). The 277 female subjects with a VTE event have 1000 Genomes imputed genotype data. After including the 1000 Genomes imputed and genotyped SNPs, two SNPs on chromosome 7p13 (rs10215876 and chr7:44909852:D) at 5-6kb 3’ of the *PURB* gene (imputation quality of 0.89 and 0.87, respectively), one SNP on chromosome 9p21.2 (rs4878679) in *LINGO2* (imputation quality = 0.99), and one SNP on the X chromosome (rs2191549) in *RDXP2* at 11k 3’ downstream (imputation quality = 1.0), were found to be significantly associated with reduced hazards of pregnancy-related VTE (HR = 0.40, 95% CI (0.28–0.58), *p* = 1.15 × 10^−8^; HR = 0.41, 95% CI (0.29–0.58), *p* = 3.34 × 10^−8^); HR = 0.63, 95% CI (0.52–0.75), *p* = 3.31 × 10^−7^; and HR = 0.52, 95% CI (0.40-0.69), *p* = 4.92 × 10^−7^, respectively) (Table 3). The two SNPs on 7p13 are in high linkage disequilibrium (r^2^ = 0.85). The Manhattan and the Q-Q plots are depicted in Figure 1. All of the top SNPs are included within the confidence interval, indicating that these SNPs are true-positives. The genomic inflation factor was 1.02, suggesting no evidence of population stratification in our study. The top 20 SNPs results are shown in Supplementary Table S1.

### 3.2. Validation Studies

Since we did not have a validation data set, we performed an internal cross-validation study and an internal meta-analysis for the top four SNPs.

**Figure 2:**
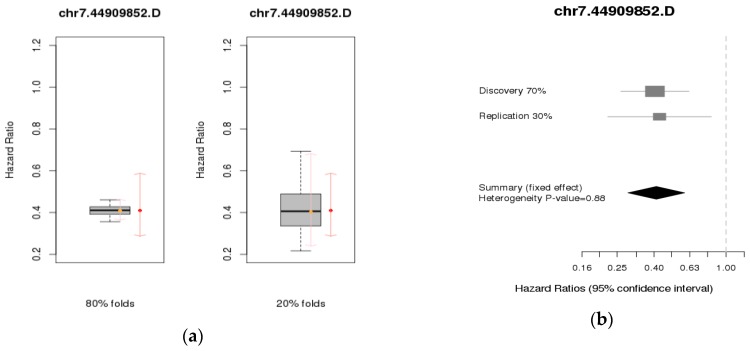
(**a**) Internal cross-validation using 80% and 20%, and (**b**) Internal meta-analysis using 70% of the data as discovery and 30% as replication for chr7.44909852.D.

For the internal cross-validation, we used the HR distribution over 45 replicates. These replicates were created by sorting the residuals of the Cox model with no covariates and counting off groups of 10 that created 10 folds with balanced time-to-event information. There are 45 ways of choosing 2 folds out of the 10 for the 20% test set, resulting in the 80% and 20% data sets having 45 combinations each. The estimated HRs for the 80% and 20% replicates from cross-validation (C-V) had the same magnitude as the HRs from the discovery set, narrower and wider (possibly due to the small sample size) confidence intervals for 80% and 20%, respectively. On average, the internal cross-validation confirmed similar magnitudes for the HRs in the training and testing sets for *PURB* rs10215876 and chr7:44909852:D, *LINGO2* rs4878679, and *RDXP2* rs2191549. For the internal meta-analysis, we used the HRs for the discovery and replication sets. There is no evidence of heterogeneity of the HRs between the two sets. The meta-analysis results are shown in Table 4. The results of the two validation results are depicted only for the SNP chr7:44909852:D, since it is in linkage disequilibrium with rs10215876 in Figure 2. The results for the other three SNPs are shown in the Supplementary Materials (Supplementary Figure S3A,B).

### 3.3. Gene Expression

**Figure 3:**
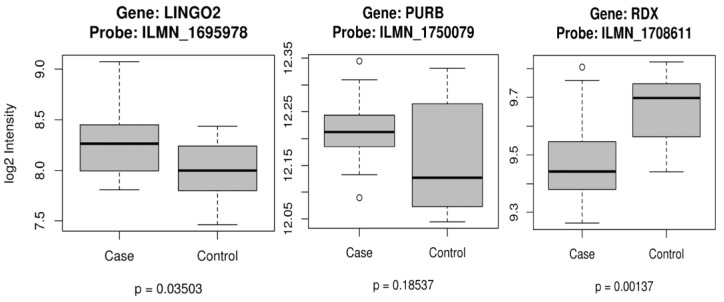
Gene Expression for females with VTE and controls for three genes: *LINGO2*, *PURB*, and *RDX*.

For the differential gene expression analysis, we use only females, 22 VTE and 8 controls. There is one expression probe in each gene in our dataset. We used the RDX gene on chromosome 11 as a surrogate gene for RDXP2 (a pseudogene for RDX on chromosome X). The boxplots for LINGO2 (probe:ILMN_1695978), PURB, and RDX are depicted below. From those three genes, only PURB was not significant (Figure 3).

### 3.5. Cox Proportional Hazards Model

The Cox proportional hazards model assumes a hazard function in the form

(1) $$\lambda_{i}\left(t\right)=\;\lambda_{0}\left(t\right)e^{X_{i}\left(t\right)\beta }$$

where $\lambda_{0}\left(t\right)$ is an unspecified non-negative function of time. The counting process formulation replaces the pair of variables $\left(T_{i},\;\delta_{i}\right)$ with the pair of functions $\left(N_{i}\left(t\right),\;\delta_{i}\left(t\right)\right),$ where $N_{i}\left(t\right)$ is the number of observed events in [0, *t*] for unit *i*, and $Y_{i}\left(t\right)$ is 1 if unit *i* is under observation and at risk at time *t* and 0 otherwise [26]. In our data, being pregnant at the time of a VTE is an event ($Y_{i}\left(t\right)$ = 1) and having a VTE but not being pregnant at the time of the VTE is a non-event ($Y_{i}\left(t\right)$ = 0). Pregnancy (9 months) or pregnancy/postpartum (12 months) unrelated to VTE is a time-dependent risk factor. Pregnancy or pregnancy/post-partum starts and stops. We use the R library ‘survival’ and the ‘coxph’ function to perform the analysis. The R code used for the analysis is included in the Supplementary Material.

The statistical models used for this analysis in R are:

model.null ≤ coxph(Surv(start, stop, VTEevent) ~ pregnancy + stroke/MI + MN state)

model.full ≤ coxph(Surv(start, stop, VTEevent) ~ pregnancy + stroke/MI + MN state + SNP)

The effect of the SNP is assessed using the likelihood ratio test (LRT) with 1 degree of freedom, by comparing the two models described above.

## 4. Discussion

In this first time-to-event genome-wide association analysis of pregnancy-related VTE, we identified the novel intronic SNPs *PURB* chr7.44909852.D and rs10215876, *LINGO2* rs4878679, and *RDXP2* rs2191549 (X chromosome) as being associated with significantly decreased hazard ratios. We used a (start, stop) counting process input in a Cox regression analysis taking into account the number of pregnancies. An event was defined as pregnancy-related VTE, and we adjusted for pregnancy, stroke/MI, and Minnesota state of residence. This model has been used mostly in clinical trials when the investigators would like to know the effect of a candidate gene’s genetic variants and a risk factor-related outcome during a treatment therapy, and rarely has been applied in a whole genome association analysis [32].

There are several limitations to our study. The first is that our study population was a referral population. Thus, the women who were in the risk set but whose VTE was after age 45 may include a higher proportion of women who never used oral contraceptives or hormone replacement, e.g., such previous oral contraceptive or hormone replacement users chose not to come to Mayo Clinic. Likewise, since we are unable to adjust for duration of exposure to oral contraceptive use or hormone replacement therapy, we are unable to assess for a potential ‘depletion of susceptibles’ bias [33]. However, we note that our estimate for the hazard of VTE with hormone replacement therapy is well within the 95% confidence intervals of other studies assessing the risk of VTE (both current use and first use within the 1st year) [34]. Our estimate of the hazard of VTE for oral contraceptive use was higher than the risk of newer formulations (range of pooled analyses: odds ratios from 2.38 to 5.13 with the 95% confidence interval for the latter just barely excluding our hazard ratio of 9.68 (7.48 to 12.52) [35]. Since our samples are from a referral study, the percentage of oral contraceptives used at a VTE event (37.2%) is more than expected based on previous case-cohort studies. Furthermore, the incidence of pregnancy-related VTE is 1–2 per 1000 pregnancies [5]. In our sample of 634 VTE women, 30 women had pregnancy-related VTE. Although we applied a time-to-event analysis, using Cox proportional hazards for the event (pregnancy-related VTE), the small number of events (*N* = 30) limits our inference. However, because VTE in pregnancy is a relatively rare event, a sample of 15,000 to 30,000 pregnancies would be needed to obtain an equivalent number of 30 pregnancy-related VTE events, suggesting that our initial results would be useful for future investigation of pregnancy-related VTE. The last weakness is the lack of a replication study. Unfortunately, none of the VTE cohorts known to us had information on pregnancy-related VTE, since these cohorts focused mainly on an idiopathic VTE event. Since we could not validate our findings in another data set, we performed an internal cross-validation and an internal meta-analysis to confirm our findings. We recognize that these approaches are complementary and not a replacement for a real validation using a similar statistical method and study design. Thus, a validation in an independent European ancestry population is needed.

The four SNPs associated with pregnancy-related VTE show a specific genetic background in the European population. The *PURB* chr7.44909852.D and rs10215876 SNPs have the minor allele TTTA allele frequency of 9–11% and minor allele T of 12% in the European population, respectively. The SNP rs10215876 has the alternate allele as the minor allele in the African and East Asian populations, 45% and 28%, respectively. The *LINGO2* rs4878679 SNP has an A (minor) allele frequency of 37% in the European population, 16% in the African population, and 47% in the South Asian population. In the East Asian population, the alternate allele is the minor allele with a frequency of 36%. The *RDXP2* rs2191549 SNP has a G (minor) allele frequency of 12–13% in the European population, 27% in the African and East Asian populations, and 7% in the South Asian population [36].

The *LINGO2* SNP identified in our gene-environment (G-E) was also identified in our G-E genome-wide association (GWA) analysis for the use of hormone replacement therapy three months prior to the VTE event. In that analysis, we applied a gene-environment case-only approach using logistic regression and a likelihood ratio test with 1 degree of freedom [37]. The model was adjusted for age and Minnesota state of residence. The most significant result was in the *LINGO2* rs10969259 with a G-E Odds Ratio (OR_gxe)_) = 2.02, with *p-value* = 4.93 × 10^−7^) [38]. The *LINGO2* SNPs rs4878679 and rs10969259 are 1.49 Mb apart and in linkage equilibrium in the European population from Phase 3 of the 1000 Genomes Project (R-squared = 0.003, D’ = 0.1).

The *PURB* (Purine-Rich Element Binding Protein B) gene product is a sequence-specific, single-stranded DNA-binding protein. It binds preferentially to the single strand of the purine-rich element, PUR, which is present at origins of replication and in gene-flanking regions in a variety of eukaryotes from yeasts through humans. Thus, it is implicated in the control of both DNA replication and transcription. Deletion of this gene has been associated with myelodysplastic syndrome and acute myelogenous leukemia, both of which are associated with VTE [16]. *LINGO2* (Leucine Rich Repeat and Ig Domain Containing 2) is a protein-coding gene. Recently, *LINGO2* was found to be associated with endometrial cancer [39]. *RDXP2* is a pseudogene located on Xp21.3, and it is a truncated version of the *RADXIN* gene located on 11q23. Using the comparative Toxicogenomics database, acetaminophen affects the expression of RDX mRNA, and ethinyl estradiol results in a decreased expression of RDX mRNA as well as a decreased expression of RDX protein. Furthermore, thrombophilia and thrombosis are related via ethinyl estradiol [40]. These findings have brought new insights into the mechanism of pregnancy-related VTE, and these results will help further investigation via functional studies and pathways analysis. We have also provided results on how these genes expressed in a group of women with VTE compared with women controls (Figure 3). We also recognize that functional validation and follow-up studies are needed.

## 5. Conclusions

This study is one of the first studies to use time-to-event to identify genetic variants to pregnancy-related VTE. Usually, investigators have compared women with VTE, with and without pregnancy, to identify genetic risk factors related to pregnancy-related VTE using a case-control design or comparing women with VTE to women without VTE using a logistic regression model or using chi-square tests in contingency tables. Our objective is to understand what mechanisms make some women have VTE during pre-and postpartum and some not. Using our results, we have also checked whether the usual genetic variants identified in VTE cases are also seen in these sets of women with pregnancy-related VTE. None of the main genetic risk factors, such as Factor V Leiden (*FVL*), Factor II (*FII*), or *ABO* genes, were significant in our analysis (Supplementary Table S3), suggesting that the genetic variants associated with pregnancy related-VTE may be not the same as the known idiopathic VTE genetic risk factors. We recognize that more work is needed to confirm these findings through replication using a similar study design, functional validation, and follow-up studies.

## Figures and Tables

**Table 1:** (**a**) Distribution of main venous thromboembolism (VTE) risk factors in these 633 females; (**b**) Distribution of pregnancies between females, and within the pregnant women with VTE events, including age at enrollment in the study and age at VTE, stroke/myocardial infarction (MI), and state of residence.

**(a)**			
**Risk Factors**	***N* = 633**	HR * (95% CI)	***p*-Value**
Pregnancy			
Median (Q1, Q3)	0 (0, 2)	3.30 (2.24, 4.88)	1.85 × 10^−9^
Oral Contraceptive ^§^			
Yes ( *N*, %)	103, 16.3%	9.68 (7.48, 12.52)	<2 × 10^−16^
No ( *N*, %)	530, 83.7%	REF	
Hormone Replacement Therapy ^§^			
Yes	14, 2.12%	3.17 (1.84, 5.46)	3.38 × 10^−5^
No	619, 97.8%	REF	
Family History of VTE			
Yes	216, 33.5%	(0.78, 1.29)	0.983
No	373, 58.9%	REF	
Missing	44, 6.95%		
**(b)**			
**Variables**	**VTE during Pregnancy (*N* = 30)**	**VTE not during Pregnancy (*N* = 75)**	**VTE and Never Pregnant (*N* = 172)**
Age at enrollment, years (SD)	40.80 (16.24)	40.32 (9.90)	36.25 (11.46)
Age at VTE, years (SD)	27.67 (6.20)	34.91 (6.70)	30.77 (9.01)
Stroke/MI, *N* (%)	2 (6.67)	5 (6.67)	19 (11.05)
Minnesota resident, *N* (%)	14 (46.67)	30 (40.00)	70 (40.70)
Non-Minnesota resident, *N* (%)	16 (53.33)	45 (60.00)	102 (59.30)

* The Hazard Ratio (HR) and *p*-value are from the multivariate Cox Proportional Hazard (CoxPH) statistical model adjusted for Stroke/MI and Minnesota state of residence. ^§^ Oral contraceptive and Hormone Replacement Therapy are at the time of the VTE event. REF: represents the VTE women that did not take Oral Contraceptive or Hormone Replacement Therapy or did not have family history of VTE. SD: Standard Deviation.

**Table 2:** Distribution of pregnancies between females and within the pregnant women with VTE events, how many females have VTE at 1st pregnancy, 2nd pregnancy, and 3+ pregnancy (meaning either the 3rd, or 4th or 5th pregnancy and so on).

Variables	VTE during Pregnancy 1 (*N* = 18)	VTE during Pregnancy 2 (*N* = 7)	VTE during Pregnancy 3+ (*N* = 5)	VTE not during Pregnancy (*N* = 75)	VTE and Never Pregnant (*N* = 172)
Age at enrollment, years (SD)	39.11 (16.89)	44.00 (18.82)	42.40 (11.55)	40.32 (9.90)	36.25 (11.46)
Age at VTE, years (SD)	25.08 (5.23)	29.08 (4.71)	35.02 (5.30)	34.91 (6.70)	30.77 (9.01
Stroke/MI, *N* (%)	1 (5.56)	1 (14.29)	0 (0.00)	5 (6.67)	19 (11.05)
Minnesota resident, *N* (%)	8 (44.44)	3 (42.86)	3 (60.00)	30 (40.00)	70 (40.70)
Non-Minnesota resident, *N* (%)	10 (55.56)	4 (57.14)	2 (40.00)	45 (60.00)	102 (59.30)

**Table 3:** Summary of the Cox regression model for the covariates only (null model) and including the four top single nucleotide polymorphisms (SNPs) separately.

Caption	Characteristic	Hazard Ratio (95% Confidence Interval)	Standard Error	*p*-Value
Multivariable model (null)	Pregnancy	3.19 (2.16, 4.71)	0.20	3.56 × 10^−7^
	Stroke/MI	0.49 (0.33, 0.74)	0.21	1.65 × 10^−4^
	Minnesota resident	0.84 (0.66, 1.07)	0.12	0.162096
Multivariable model Chr7:rs10215876	Pregnancy	3.21 (2.17, 4.74)	0.20	3.16 × 10^−7^
	Stroke/MI	0.47 (0.32, 0.71)	0.21	6.41 × 10^−5^
	Minnesota resident	0.83 (0.65, 1.06)	0.12	0.130
	Chr7: rs10215876	0.40 (0.28, 0.58)	0.18	1.15 × 10^−8^
Multivariable model Chr7:44909852.D	Pregnancy	3.23 (2.18, 4.77)	0.20	2.78 × 10^−7^
	Stroke/MI	0.47 (0.32, 0.71)	0.21	6.45 × 10^−5^
	Minnesota resident	0.83 (0.66, 1.06)	0.12	0.134
	Chr7.44909852.D	0.41 (0.29, 0.58)	0.18	3.34 × 10^−8^
Multivariable model Chr9:rs4878679	Pregnancy	3.11 (2.10, 4.59)	0.20	5.85 × 10^−7^
	Stroke/MI	0.48 (0.32, 0.72)	0.21	8.19 × 10^−5^
	Minnesota resident	0.80 (0.63, 1.02)	0.12	0.0709
	Chr9:rs4878679	0.63 (0.52, 0.75)	0.09	3.31 × 10^−7^
Multivariable model ChrX:rs2191549	Pregnancy	3.26 (2.21, 4.82)	0.20	2.24 × 10^−7^
	Stroke/MI	0.49 (0.33,0.74)	0.21	1.81 × 10^−4^
	Minnesota resident	0.83 (0.65,1.05)	0.12	0.150
	ChrX: rs2191549	0.52 (0.40, 0.69)	0.14	4.92 × 10^−7^

**Table 4:** Results of the meta-analysis.

SNP	CHR	HR	95% CI	Heter_Pvalue	Meta_Pvalue
rs10215876	7	0.410	(0.288, 0.584)	0.615	7.49 × 10^−7^
chr7.44909852.D	7	0.412	(0.287, 0.590)	0.883	1.41 × 10^−6^
rs4878679	9	0.628	(0.522, 0.754)	0.587	6.49 × 10^−7^
rs2191549	23	0.516	(0.392, 0.679)	0.983	2.29 × 10^−6^

CHR: chromosome; HR: Hazard Ratio; Heter=heterogeneity; Meta=meta-analysis.

## Supplementary Materials

The following are available online at http://www.mdpi.com/1660-4601/14/10/1228/s1, Table S1: The top 20 SNPs results with INFO metric > 0.8 and MAF > 0.005, Table S2: Replication of females only results from Heit et al. 2011 using our VTE due to pregnancy results, Table S3: Results of the known SNPs for VTE in general in women with pregnancy-related VTE, Figure S1: Pregnancy Distribution for 634 women with VTE, Figure S2: Locus zoom plots for the three top SNPs and genes: *PURB*, *LINGO2* and *RDXP2*, Figure S3: Results for the internal cross-validation (C-V) and meta-analysis (M-A) for the top 3 SNPs, chr7:rs10215876, chr9:rs4878679, and chrX:rs2191549.
