# Supplementary material for: Identification of Genetic Interaction with Risk Factors Using a Time-To-Event Model
Source: Int J Environ Res Public Health. 2017 Oct 15;14(10):1228. doi: 10.3390/ijerph14101228 (PMC5664729; doi:10.3390/ijerph14101228)

Supplementary Tables:

Supplementary Table 1: The top 20 SNPs results with INFO metric > 0.8 and MAF > 0.005.

SNP	CHR	Base Pair Position	GENE	Minor Allele	Minor Allele Frequency	Hazard Ratio (95% CI)	Standard Error	P-value	INFO metric*
rs10215876	7	44910432	PURB	T	0.1238	0.4 (0.28,0.58)	0.1797	1.15E-08	0.899
chr7:44909852:D	7	44909852	PURB	TTTA	0.1162	0.41 (0.29,0.59)	0.1835	3.33E-08	0.87
rs4878679	9	28079489	LINGO2	A	0.4021	0.63 (0.52,0.75)	0.0934	3.31E-07	0.993
rs146785149	9	28091543	LINGO2	A	0.4044	0.63 (0.53,0.76)	0.0928	4.59E-07	0.999
rs2191549	23	27522117	RDXP2	G	0.1667	0.52 (0.4,0.69)	0.1388	4.91E-07	1
rs112665906	10	5884478	TRNAV26	A	0.0138	5.74 (3.27,10.07)	0.2869	7.55E-07	0.809
rs2331173	7	44914229	PURB	C	0.1048	0.45 (0.32,0.65)	0.1811	8.38E-07	0.985
rs9775023	9	28091725	LINGO2	A	0.3599	0.63 (0.52,0.76)	0.0971	9.33E-07	0.99
rs7847672	9	28093874	LINGO2	A	0.3673	0.63 (0.52,0.76)	0.0958	1.07E-06	1
rs184940211	10	19102071	LOC645120	T	0.0073	11.51 (5.48,24.15)	0.3782	1.11E-06	0.869
rs66529353	23	27521976	RDXP2	A	0.1502	0.52 (0.39,0.69)	0.146	1.21E-06	0.976
rs189318632	10	19073144	LOC645120	C	0.0063	13.63 (6.28,29.6)	0.3956	1.23E-06	0.811
rs7061554	23	27518847	RDXP2	G	0.1502	0.52 (0.39,0.69)	0.146	1.23E-06	0.975
rs57479682	23	27517013	RDXP2	T	0.1501	0.52 (0.39,0.69)	0.146	1.24E-06	0.975
rs58717083	23	27521647	RDXP2	G	0.1503	0.52 (0.39,0.69)	0.146	1.26E-06	0.975
rs6630485	23	27497228	SMEK3P	C	0.1484	0.52 (0.39,0.69)	0.146	1.26E-06	0.976
rs57704983	23	27521134	RDXP2	A	0.1503	0.52 (0.39,0.69)	0.146	1.26E-06	0.975
rs6418592	23	27519496	RDXP2	G	0.1503	0.52 (0.39,0.69)	0.146	1.27E-06	0.975
chrX:27532240:I	23	27532240	RDXP2	AT	0.1489	0.52 (0.39,0.69)	0.1475	1.28E-06	0.971
rs6971158	7	44918206	PURB	C	0.1063	0.47 (0.33,0.66)	0.1774	1.33E-06	0.991

*INFO metric = a statistical information metric, which is highly correlated with the squared correlation metrics output by BEAGLE and MACH imputation software. Values range from 0 to 1, where 1 means no uncertainty in the imputed genotypes. This is not based only on female subjects with VTE; it is based on all subjects with imputed data.

Supplementary Table 2: Replication of females only results from Heit et al. 2011 using our VTE due pregnancy results*

SNP	CHR	Base Pair Position	GENE	Minor Allele	Minor Allele Frequency	Hazard Ratio (95% CI)	Standard Error (SE)	P-value	INFO metric*
rs6687813	1	169477574	F5	A	0.1303	1.11 (0.87,1.41)	0.1224	0.409	0.995
rs6025	1	169519049	F5	T	0.0902	1.12 (0.85,1.49)	0.1443	0.427	0.954
rs9328375	6	6592023	LY86	T	0.3006	1.08 (0.89,1.30)	0.0957	0.445	1
rs1073897	6	6592984	LY86	A	0.2475	1.10 (0.90,1.34)	0.1007	0.354	0.978
chr9:136132908:I	9	136132908	ABO	TC	0.4783	0.86 (0.72,1.03)	0.0911	0.101	0.986
rs687289	9	136137106	ABO	A	0.4639	0.86 (0.72,1.03)	0.0914	0.096	0.988
rs2519093	9	136141870	ABO	T	0.3033	0.93 (0.78,1.12)	0.0936	0.466	0.985
rs643434	9	136142355	ABO	*A*	0.4813	0.86 (0.72,1.03)	0.0911	0.099	0.998
rs505922	9	136149229	ABO	*C*	0.4652	0.87 (0.73,1.04)	0.0907	0.114	1
rs630014	9	136149722	ABO	*A*	0.3578	1.13 (0.94,1.35)	0.0906	0.189	1
rs1799963	11	46761055	F2	*A*	0.0173	0.68 (0.31,1.47)	0.398	0.296	0.861

Supplementary Table 3: Results of the known SNPs for VTE in general in women with pregnancy-related VTE

SNP	CHR	Minor Allele	Major Allele	MAF	Coef.	SE.Coef	HR	Chisq. Wald	p.wald	HR.lcl	HR.ucl	Chisq .LRT	p.LRT
rs6025	1	T	C	0.09	0.103	0.146	1.11	0.497	0.481	0.833	1.475	0.483	0.487
rs2274976	1	T	C	0.05	0.040	0.195	1.04	0.041	0.839	0.710	1.524	0.041	0.840
rs1801131	1	G	T	0.31	0.099	0.091	1.10	1.182	0.277	0.924	1.320	1.169	0.280
rs1801133	1	A	G	0.33	0.048	0.088	1.05	0.287	0.592	0.882	1.246	0.286	0.593
rs1799808	2	T	C	0.34	-0.049	0.093	0.95	0.274	0.601	0.794	1.143	0.275	0.599
rs1799809	2	G	A	0.44	0.0043	0.086	1.00	0.002	0.961	0.848	1.190	0.003	0.961
rs8176747	9	G	C	0.09	-0.060	0.145	0.94	0.174	0.677	0.709	1.251	0.177	0.674
rs8176746	9	T	G	0.09	-0.060	0.145	0.94	0.174	0.677	0.709	1.251	0.174	0.674
chr9:136132908:I	9	TC	T	0.48	-0.141	0.091	0.87	2.416	0.120	0.727	1.038	2.422	0.120
rs1799963	11	A	G	0.02	-0.355	0.397	0.70	0.799	0.371	0.322	1.527	0.888	0.346

Supplementary Figures:

Supplementary Fig. 1: Pregnancy Distribution for 634 women with VTE.

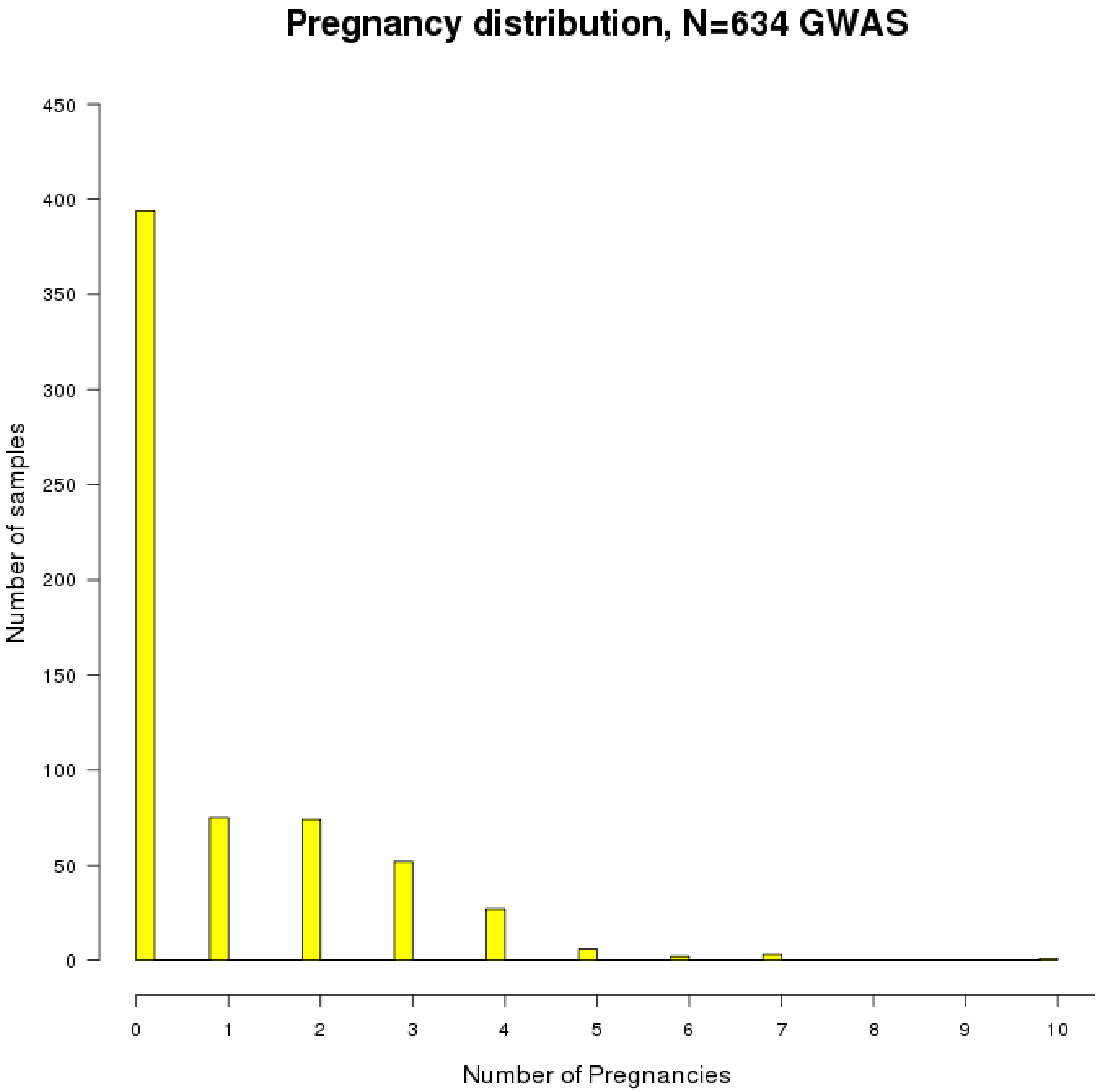

Supplementary Fig. 2: Locus zoom plots for the three top SNPs and genes: *PURB, LINGO2* and*RDXP2*.

PURB

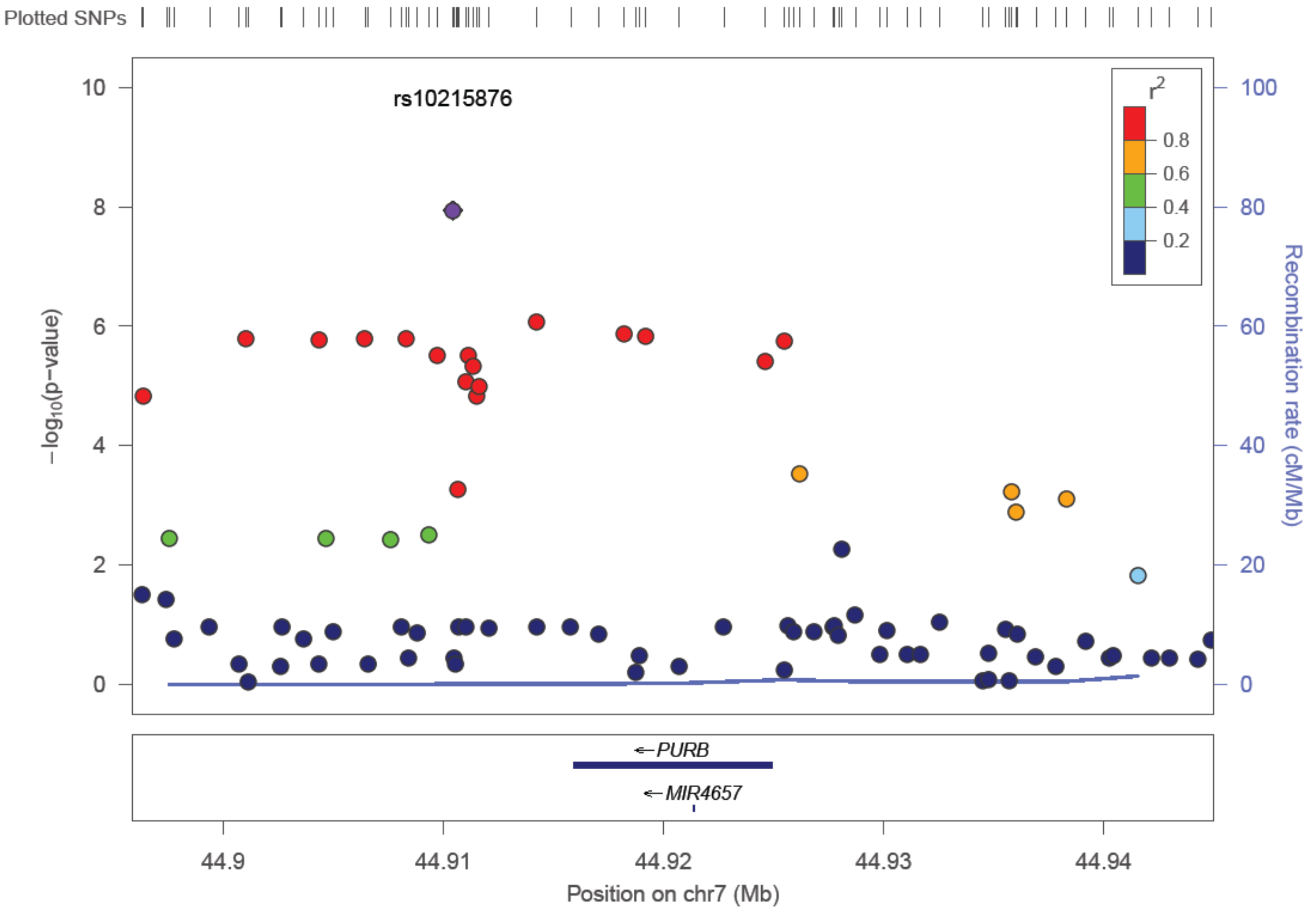

LINGO2

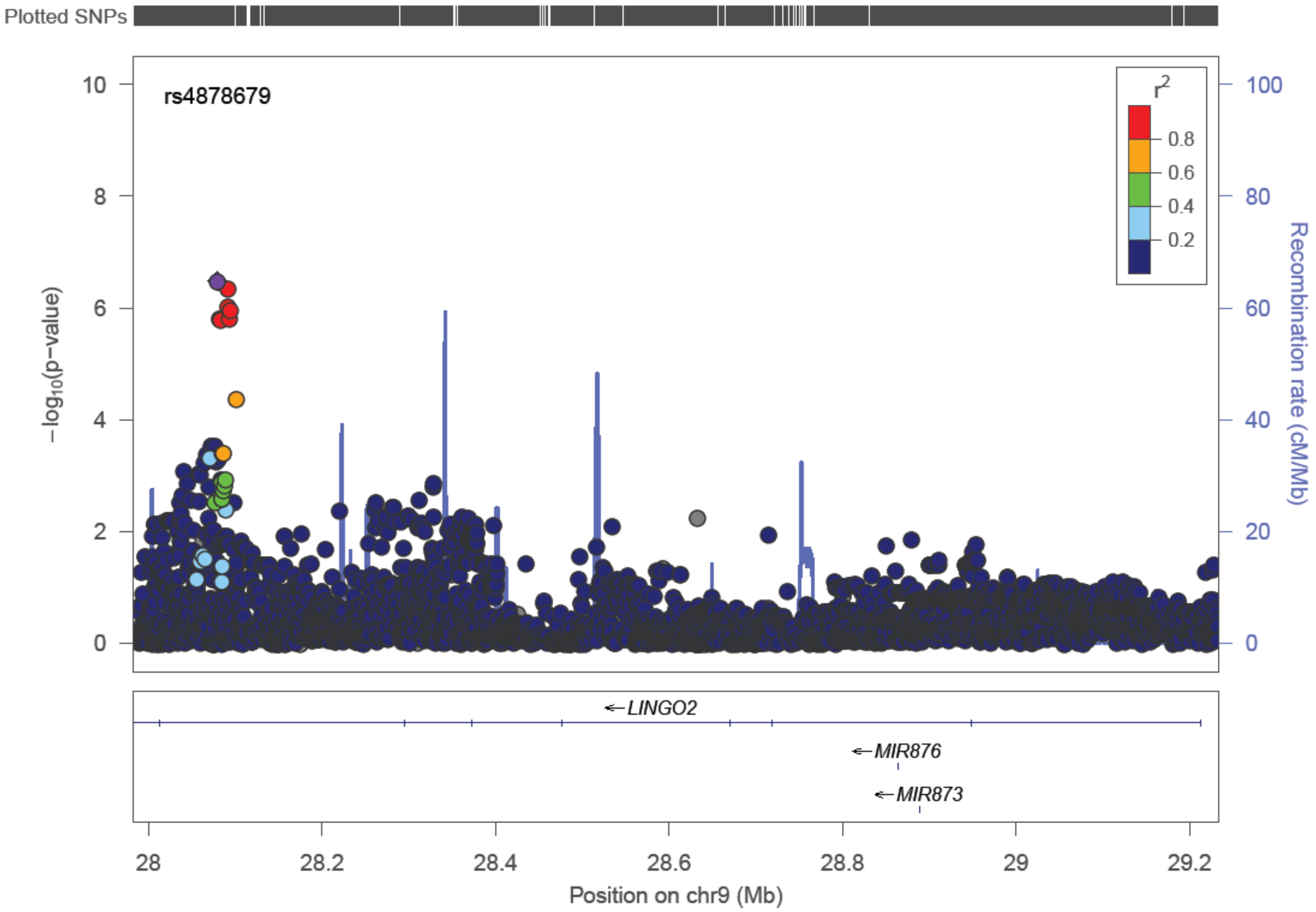

Plotted SNPs
rs4878679
r²
0.8
0.6
0.4
0.2
−log₁₀(p−value)
Recombination rate (cM/Mb)
10
8
6
4
2
0
100
80
60
40
20
0
LINGO2
MIR876
MIR873
28
28.2
28.4
28.6
28.8
29
29.2
Position on chr9 (Mb)

RDXP2

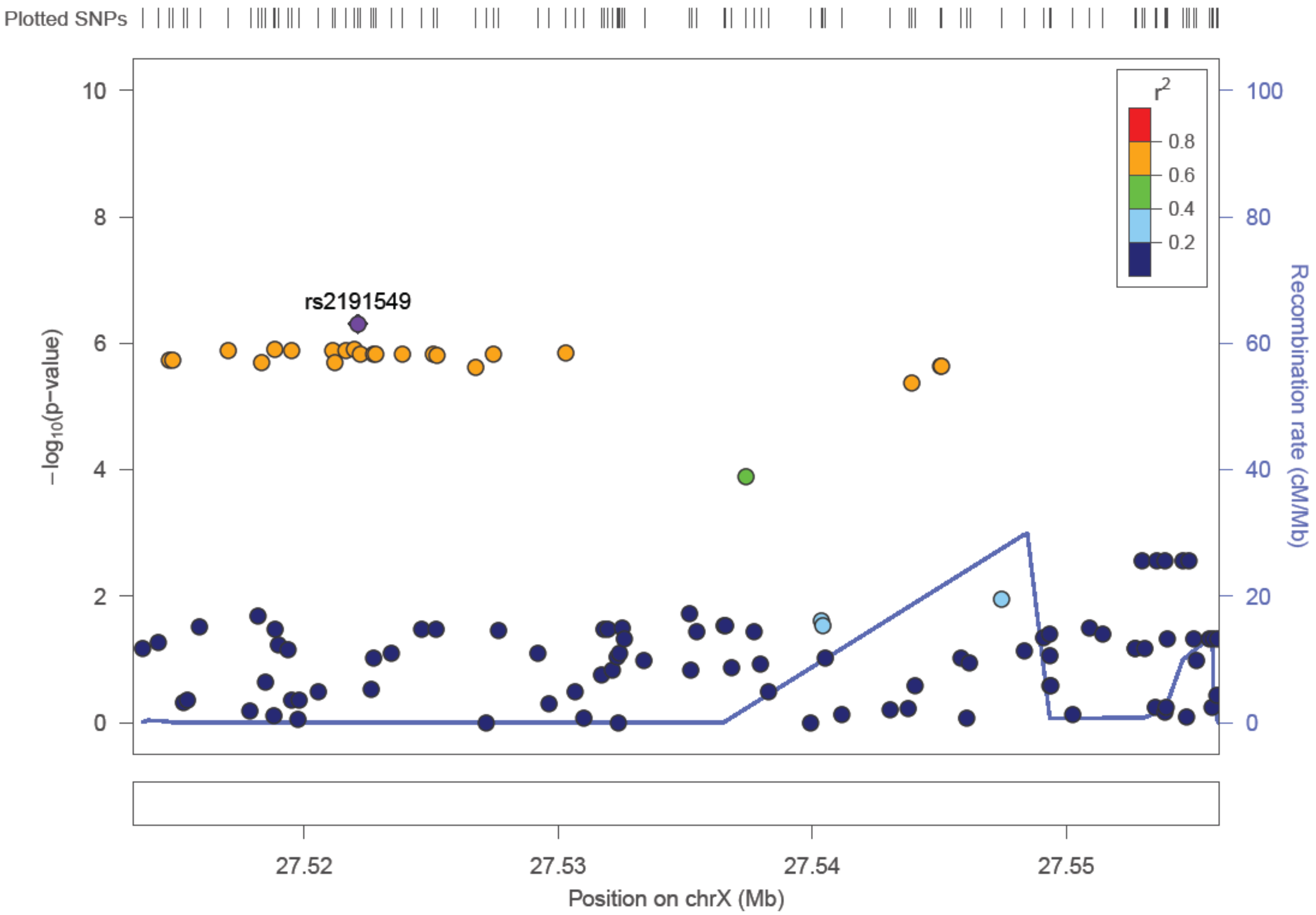
Plotted SNPs
rs2191549
r²
0.8
0.6
0.4
0.2
10
8
6
4
2
0
−log10(p−value)
100
80
60
40
20
0
Recombination rate (cM/Mb)
27.52
27.53
27.54
27.55
Position on chrX (Mb)

Supplementary Fig. 3: Results for the internal cross-validation (C-V) and meta-analysis (M-A) for the top 3 SNPs, chr7:rs10215876, chr9:rs4878679, and chrX:rs2191549.

The boxplots represent the distribution of the hazard ratios (HRs) over 45 replicates. The 45 replicates were created by sorting the residuals of the cox model without covariates, and counting off groups of 10 (the first residual goes in fold1, the 11th residual foes in fold 1, the 2nd residual foes in fold 2, and so on). This created 10 folds. There are 45 ways of choosing 2 folds out of 10 for the 20% test set, resulting in the 80% and 20% data sets to have 45 combinations each. The first confidence interval (CI) that overlaps the boxplot is the CI based on the 45 replications and the dot is the average of these replications. The second CI is the discovery analysis data using 100% of the data. The boxplot on the left and on the right represents the 80% and 20% replicates, respectively.

(A)

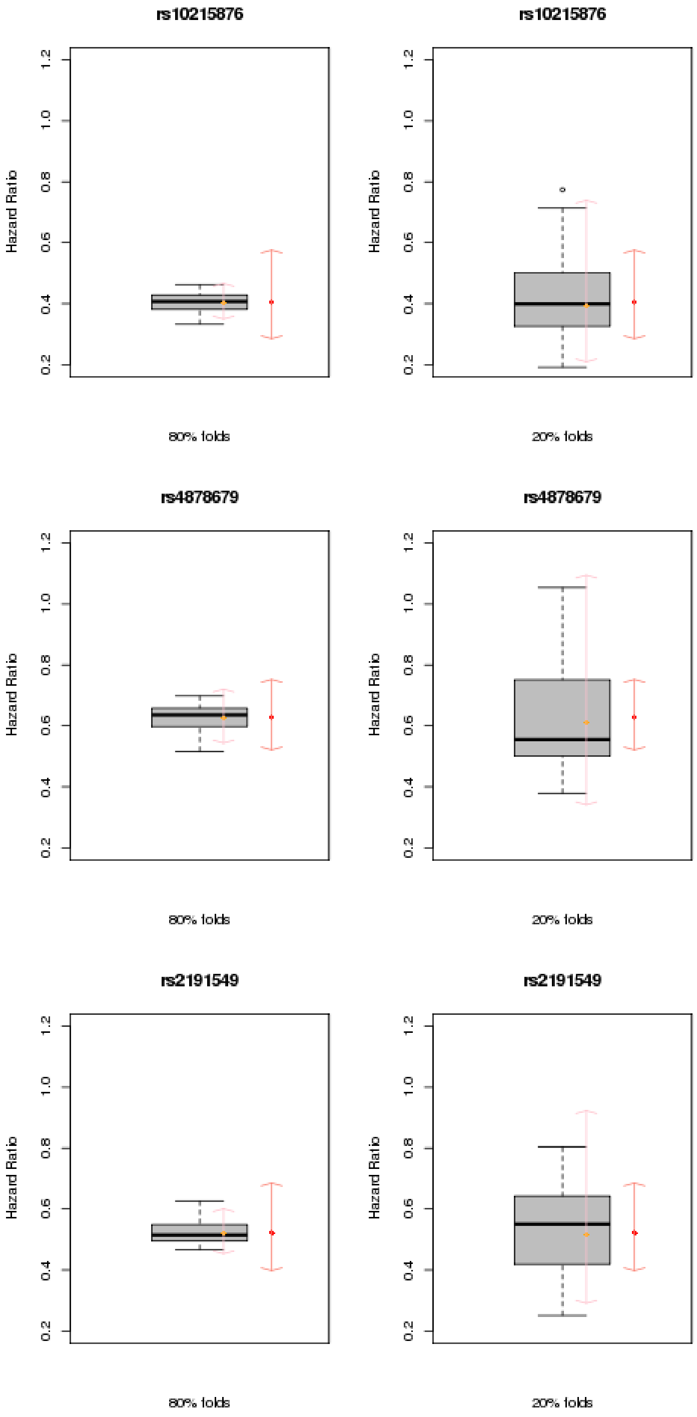

For the internal **M-A**, the following 3 plots show the discovery and validation results for chr7:rs10215876, chr9:rs4878679, and chrX:rs2191549. We applied a fixed-effect meta-analysis approach where we divided the samples in two datasets using the same strategy of sorting the residuals from the model with no covariates, as described in the main text: the first set as the discovery and consisting of 70% of the samples, and the second set as the replication and consisting of 30% of the samples. A Woolf's test of homogeneity of hazard ratios (HRs) between discovery and validation sets was performed to assess whether the distribution of HRs between the two sets is compatible with a common HR. The top and bottom vertical box plots represent the discovery and replication sets (70% and 30%), respectively. In each forest plot, the confidence interval for each set is given by a horizontal line and the point estimate is given by a square whose height is inversely proportional to the standard error of the estimate. The summary hazard ratio from the meta-analysis is drawn as a diamond with horizontal limits at the confidence limits and width inversely proportional to its standard error.

(B)

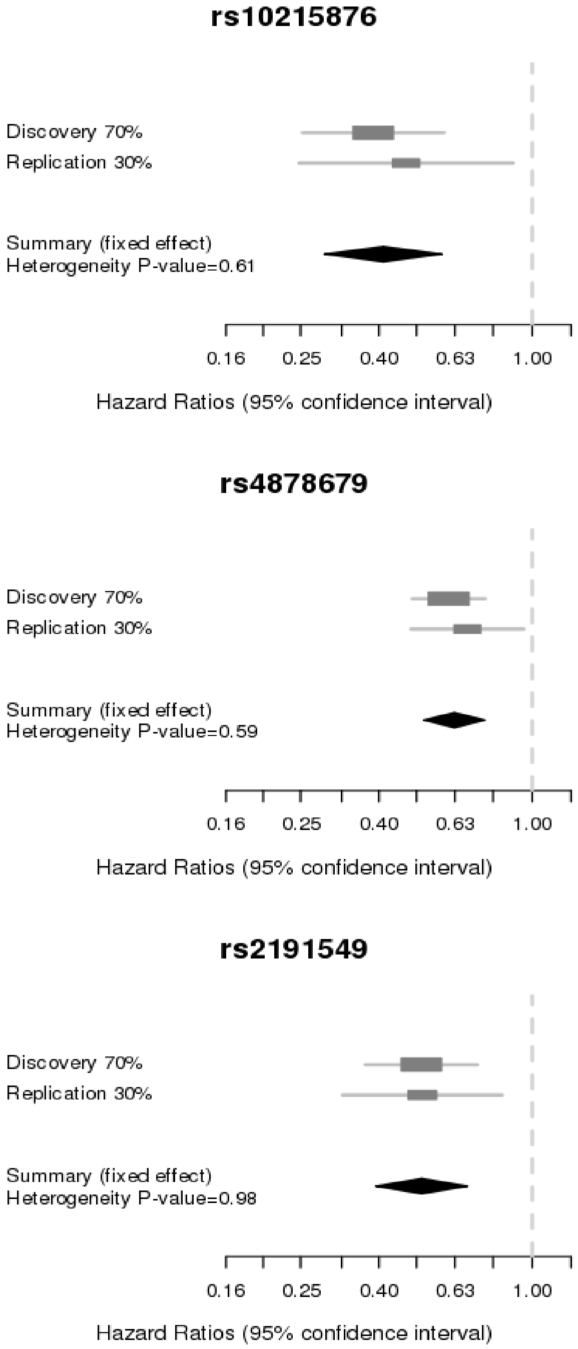

Supplement: Supplementary file 1 [file ijerph-14-01228-s001.pdf]
